# Blood-Based Biomarkers in Intracerebral Hemorrhage

**DOI:** 10.3390/jcm12206562

**Published:** 2023-10-16

**Authors:** Xin-Ni Lv, Zuo-Qiao Li, Qi Li

**Affiliations:** 1 Department of Neurology, The First Affiliated Hospital of Chongqing Medical University, Chongqing 400016, China; xinnilv@126.com (X.-N.L.); lizuo_23@163.com (Z.-Q.L.); 2 Department of Neurology, The Second Affiliated Hospital of Anhui Medical University, Hefei 230601, China

**Keywords:** biomarkers, intracerebral hemorrhage, hematoma growth, perihematomal edema, prognosis

## Abstract

Intracerebral hemorrhage (ICH) is one of the most lethal subtypes of stroke, associated with high morbidity and mortality. Prevention of hematoma growth and perihematomal edema expansion are promising therapeutic targets currently under investigation. Despite recent improvements in the management of ICH, the ideal treatments are still to be determined. Early stratification and triage of ICH patients enable the adjustment of the standard of care in keeping with the personalized medicine principles. In recent years, research efforts have been concentrated on the development and validation of blood-based biomarkers. The benefit of looking for blood candidate markers is obvious because of their acceptance in terms of sample collection by the general population compared to any other body fluid. Given their ease of accessibility in clinical practice, blood-based biomarkers have been widely used as potential diagnostic, predictive, and prognostic markers. This review identifies some relevant and potentially promising blood biomarkers for ICH. These blood-based markers are summarized by their roles in clinical practice. Well-designed and large-scale studies are required to validate the use of all these biomarkers in the future.

## 1. Introduction

Spontaneous intracerebral hemorrhage (ICH) is a common subtype of stroke and accounts for nearly 10% to 30% of all strokes [1]. ICH posed a significant health burden in China in 2019, contributing to 25 million disability-adjusted life years (DALYs). It is associated with an early mortality rate of approximately 30–40% and has shown no reduction in prevalence in recent decades [2]. The higher incidence and mortality of ICH in middle-income and low-income countries may be attributed to limited public awareness regarding preventive measures, as well as challenges in accessing healthcare services [3]. So far, few treatment possibilities have yielded conclusive benefits. However, numerous predictors of poor outcomes after ICH have been suggested, including hematoma volume, hematoma expansion, perihematomal edema (PHE), and the presence and quantity of intraventricular hemorrhage growth [4,5,6,7]. Similarly, researchers continue to search for various blood-based biomarkers to guide management and predict outcomes after acute ICH [8,9,10,11,12]. A growing number of studies have shown that many novel biomarkers, such as C-reactive protein (CRP), S100A12, and interleukin-6, are associated with adverse outcomes in patients with ICH [8,9,10]. Thus, the application of blood-based biomarkers may improve risk stratification and aid clinical decisions in patients with ICH.

Therefore, in this review, we assessed the data available in the literature regarding the diagnostic and prognostic value of circulating blood biomarkers in patients with ICH. For each biomarker, we depicted the detailed characteristics in order to shed light on potential future clinical applications.

## 2. Diagnostic Blood Biomarkers

Early administration has proven to be effective in the management of acute stroke, including intravenous thrombolysis and mechanical thrombectomy for ischemic stroke, as well as blood pressure reduction therapy for ICH. The efficacy of these treatments is highly dependent on the time from onset to administration. While prehospital treatment shows promise, it necessitates the prior differentiation of stroke subtypes. In addressing this challenge, portable computed tomography has been used; however, achieving the widespread adoption of this costly tool does not seem possible in the near future. Consequently, it is imperative that optimal blood biomarkers demonstrate enough sensitivity and are rapidly available for distinguishing between both stroke subtypes, thereby serving as a valuable diagnostic tool for acute stroke.

### 2.1. Glial Fibrillary Acidic Protein (GFAP) and Retinol-Binding Protein 4

In a recent study of 272 patients (203 ischemic strokes, 60 ICH, and 9 stroke mimics), it was shown that prehospital plasma GFAP concentrations (pg/mL) could differentiate patients with ischemic stroke from those with ICH. Furthermore, the prehospital GFAP release rate (pg/mL/minute) significantly improved the diagnostic capability of this biomarker. Of note, the median time between the last-seen-well and prehospital sample times was 50 min in this study [13]. Another study demonstrated that GFAP concentrations were associated with both hematoma volume and neurological severity in ICH patients, while no such correlation was observed in ischemic stroke patients [14]. However, in a study of 74 patients, the GFAP levels in patients with a smaller ICH volume often remained in the normal range or were only slightly elevated [15]. These findings suggest that the early release of GFAP may be influenced by the extent of hemorrhagic tissue damage following an ICH event, while it may not be influenced by the extent of tissue ischemia after ischemic stroke. Retinol-binding protein 4 (RBP-4) is a promising biomarker for distinguishing ischemic stroke from ICH. In ischemic stroke patients, RBP4 > 48.75 lg/mL and GFAP < 0.07 ng/mL were found to be independent predictors of stroke subtypes after a multivariate logistic regression analysis [16].

### 2.2. N-Terminal Pro B-Type Natriuretic Peptide (NT-proBNP)

The stroke-chip study, with a large sample size, investigated the diagnostic accuracy of 21 biomarkers for distinguishing between ischemic and hemorrhagic strokes [17]. NT-proBNP emerged as a remarkable biomarker, with a sensitivity of 44.8% and a specificity of 74.9% in its ability to differentiate between these stroke types. In a study of 189 patients (154 with ischemic stroke and 35 with ICH), it was found that ischemic stroke patients had a higher RBP-4. It was observed that ischemic stroke patients exhibited elevated levels of RBP-4, NT-proBNP, and endostatin, while they had lower levels of GFAP than ICH patients [18].

### 2.3. S100B

The S100B protein typically reaches its peak concentration on day 2 or 3 after ischemic stroke onset, whereas among ICH patients, it has been shown that there is a rapid increase in plasma S100B levels within a few hours [19]. This distinct temporal pattern allows for an early discrimination between ischemic and hemorrhagic strokes shortly after the onset of symptoms. In a previous study, among the blood samples obtained within 6 h after ictus (*n* = 337), S100B levels were significantly higher in ICH patients (107.58 vs. 58.70 pg/mL; *p* < 0.001) compared to those with ischemic stroke. The optimal concentration cut-off point for differentiation was determined to be S100B > 96 pg/mL [20].

## 3. Etiology Blood Biomarkers

### 3.1. β-Amyloid-40 and β-Amyloid-42

One study including 29 cerebral amyloid angiopathy (CAA)-associated ICH patients and 21 healthy controls found higher blood β-amyloid-40 and β-amyloid-42 concentrations in probable CAA patients than in control individuals [21]. However, a previous analysis showed contradicting results [22]. These discrepancies may be attributed to differences in the methodology used in the identical detection of antibodies. Together, these findings highlight the potential of body fluid biomarkers as preclinical indicators of CAA, and they suggest that circulating β-amyloid peptides may serve as a valuable marker for assessing microvascular damage.

### 3.2. Matrix Metalloproteinases (MMPs)

A recent study of plasma matrix MMPs showed that MMP-2 and MMP-9 are highly secreted and expressed in hemorrhagic regions in CAA, no differences were observed in plasma levels between CAA patients and healthy controls [23]. In this study, it was observed that the expression was related to endothelial cells and reactive astrocytes surrounding vessels with CAA, whereas MMP-9 expression was restricted to inflammation cells.

## 4. Prognostic Blood Biomarkers for Hematoma Expansion

### 4.1. Serum Calcium Level

Some studies have investigated the effect of admission calcium levels on hematoma growth [24,25,26,27,28]. The definition of hypocalcemia varied across the studies. A retrospective study including 1262 patients reported an association between lower mean calcium levels on admission and hematoma growth in ICH patients [24]. Two other prospective observational cohort studies also demonstrated that lower calcium levels at presentation are associated with hematoma growth in ICH [25,26]. Another study showed an association between ionized calcium < 1.16 mmol/L and hematoma growth [27]. Finally, a large sample size study screening 2103 patients reported that a higher plasma calcium level (calcium > 8.4 mg/dL) on admission was related to a reduced risk of hematoma growth when adjusted for other confounders [28]. Overall, these results suggest a compelling hypothesis that calcium may indeed play a role in the pathophysiology of ICH, with an impaired coagulation cascade serving as a potential biological mechanism.

### 4.2. Serum Magnesium Level

A retrospective cohort study found an association between lower levels of admission serum magnesium levels and hematoma growth in ICH patients. Lower admission magnesium levels were related to larger baseline hemorrhage sizes, hematoma growth, and 3-month outcomes [29]. Conversely, Goyal et al. concluded that higher serum magnesium levels were independently associated with a lower baseline hematoma size and a lower ICH score. This study found no effect of the baseline plasma magnesium level on hematoma growth [30]. The two studies diverged in their methodologies. The former study included ICH patients with underlying coagulopathy who received hemostatic treatment, which could potentially confound the impact of magnesium on hemostasis. Taken together, magnesium’s potential role in coagulation and platelet function holds promise for ICH treatment.

### 4.3. Low-Density Lipoprotein Cholesterol (LDL-C)

A previous cohort study suggested that lower LDL-C levels independently predict hematoma growth, early neurological deterioration, and 90-day outcomes after ICH [31]. Recently, several studies have reported that low levels of LDL-C were associated with an increased risk of ICH [32] and hematoma growth [26,32,33,34]. However, the pathophysiological mechanism of LDL-C in relation to ICH remains unclear. Some hypotheses suggest that reduced cholesterol levels may weaken the endothelium, potentially increasing susceptibility to arterial fragility or delayed repair following cerebral microbleeds [31,32,33,34].

### 4.4. Apolipoprotein E Genotype

In a study including 510 patients with primary ICH, it was found that lobar ICH patients who possessed apolipoprotein ε2 were at an increased risk of hematoma growth in probable or definite CAA-related ICH [35].

## 5. Blood Biomarkers for Inflammation and Perihematomal Edema

### 5.1. Neutrophils, Lymphocytes, and Neutrophil–Lymphocyte Ratio (NLR)

Blood-derived inflammatory cells have been strongly recognized as contributors to ICH-mediated secondary brain damage. Accordingly, neuroinflammation plays a critical role in the physiopathological mechanism of perihematomal edema. Neutrophils are the first leukocyte subtype that infiltrates into the hemorrhagic brain and initiates inflammatory reactions [36,37,38,39]. Perihematomal tissue obtained from ICH patients showed that low numbers of neutrophils occurred in less than 8 h after symptom onset [36]. Neutrophil infiltration was observed within 2 days and still manifested at 12 days in a postmortem study of spontaneous ICH patients who survived for 1–12 days after symptom onset [40]. In blood samples from 113 ICH patients, a lower number of neutrophils was associated with a better outcome [41]. Moreover, a large cohort including 1302 patients reported that a higher neutrophil count was associated with a reduced risk of hematoma expansion [11]. A multitude of pathways associated with neutrophils-induced neurotoxicity, including the secretion of cytokines, such as tumor necrosis factor alpha (TNF-α), interleukin-1beta and interleukin-6, chemokines, free radicals, and other toxic chemicals. These factors ultimately lead to mitochondrial dysfunction, cerebral edema, blood-brain-barrier (BBB) breakdown, and neuronal cell death [38,42,43]. Inflammatory biomarkers were most commonly explored. We found several studies investigating the predictive ability of three blood-borne leukocytes, namely lymphocytes, neutrophils, and monocytes, and the neutrophil–lymphocyte ratio (NLR). NLR was reported as the ratio of absolute neutrophils divided by the absolute lymphocytes and is commonly considered a readily available and inexpensive biomarker to measure systemic inflammation. A few studies have found that increased NLR values are associated with unfavorable outcomes [12,44,45] and perihematomal edema growth [46]. A retrospective study of 177 ICH patients showed that higher neutrophils, lower lymphocytes, and a higher NLR level on admission were independently associated with a poor 3-month outcome [12]. In a study of 213 ICH patients, the authors found a similar association between a higher NLR level and lower lymphocyte counts and unfavorable outcomes at 3 months [45], while one study showed an independent association between early neurological deterioration and an increased risk of in-hospital mortality, but not poor 3-month outcome [44]. This result is consistent with other data: Several research groups did not identify an independent association between a higher white blood cell count, neutrophils, or NLR and a poor outcome [47,48].

### 5.2. Monocytes

Several clinical studies have tested the association of monocytes with outcomes after ICH [11,47,49,50]. A recent analysis of the Genetic and Environmental Risk Factors for Hemorrhagic Stroke study demonstrated that higher monocyte counts were independently associated with greater odds of 30-day case fatality in ICH patients [49], and a larger cohort from a multi-center study reported similar findings [50]. In another large study, the results showed that a higher peripheral monocyte count was associated with a higher risk of hematoma expansion [11].

### 5.3. C-Reactive Protein (CRP)

C-reactive protein (CRP) is a classical acute-phase protein and serves as a marker of the inflammatory response featuring a homopentameric structure [51]. The relationship between admission plasma CRP and short-term fatal outcomes was explored in a small sample study, and an increase in the CRP concentration by 1 mg/L led to a 5.2% increase in the odds of first-week mortality [52]. Similarly, a previous study also pointed out that high serum CRP on admission was an independent predictor of unfavorable outcomes [53]. A multi-center study analyzed CRP concentrations and their association with ICH prognosis. Plasma CRP was assessed at admission, 24, 48, and 72 h after the onset of symptoms. Plasma CRP production elevated markedly at 48 to 72 h from admission following ICH and was independently related to poor 30-day functional outcomes. Moreover, the results also concluded that CRP > 10 mg/L independently predicted early hematoma growth and early neurological worsening, both of which were related to increased mortality [8]. Further, another study identified that the addition of CRP concentration to the ICH score significantly improved the accuracy by about 8% [54]. In conclusion, elevated levels of CRP were associated with a higher risk of poor outcomes in ICH patients and were shown to be an independent predictor of mortality [55,56].

### 5.4. S100A12

S100A12 belongs to the S100 family of calcium-binding proteins, which has newly been identified as a receptor for advanced glycation end products-binding protein. S100A12 triggers inflammatory reactions by promoting cellular and immune signaling pathways [57]. A recent study of 101 patients verified the correlation of serum S100A12 levels with hemorrhagic severity and functional outcomes in patients with acute ICH [9]. Another clinical study showed that serum S100A12 levels were correlated with NIHSS score, ICH volume, and short-term mortality after ICH [58].

### 5.5. S100B

S100B is a well-established biomarker that is associated with both brain injury and BBB dysfunction. A small case-control study stated that serum S100B had significantly raised after symptom ictus until day 3 in ICH patients compared to controls. This study has shown that there is a correlation between plasma S100B levels and 1-week mortality [59]. Similarly, another study showed that an increased serum level of S100B was closely correlated to the baseline hematoma volume and predicted poor outcome after ICH [19]. In conclusion, elevated S100B levels have been observed following ICH and may potentially contribute to the inflammatory processes associated with ICH in relation to a poorer clinical outcome.

### 5.6. High Mobility Group Box-1 (HMGB-1)

A single-center study of 65 patients depicted a significant association of HMGB-1 with levels of interleukin-6 and TNF-α and the 10-day National Institutes of Health Stroke Scale score (r = 0.845). This study also concluded that HMGB-1 levels increased dramatically in ICH patients and were associated with disease severity [60]. Serum HMGB1 level was associated with the severity of neurological impairment, indicating that HMGB1 levels could serve as a potential indicator of the extent of brain damage.

### 5.7. Pentraxin 3 (PTX3)

In a study including 307 ICH patients, it was shown that pentraxin3 (blood samples were drawn within 24 h after ictus) tends to increase after symptom onset. Additionally, high levels of pentraxin3 in ICH patients were correlated with increased mortality during the 5-year follow-up period [61].

### 5.8. Gelsolin

Plasma gelsolin plays a pivotal role in the removal of actin filaments released from deceased cells into the bloodstream [62]. Furthermore, it has the capacity to bind to a diverse range of proinflammatory and bioactive molecules that act as mediators in neurological development. In a small case-control study group, comparing ICH patients to healthy controls, a decreased concentration of admission serum gelsolin was suggested to be a valuable marker for predicting 6-month poor outcome in ICH patients [62].

### 5.9. CD163

CD163 expression was recently found to be elevated from 6 to 24 h, peaking at 72 h after ICH ictus in the perihematomal area [63]. Another study concluded that acute serum soluble CD163 levels were associated with hematoma growth, PHE expansion, early or delayed neurological deterioration, and 90-day functional outcomes [64].

### 5.10. MMP-3/MMP-9

MMPs are produced by activated microglia, infiltrating inflammatory cells following brain injury. This wide distribution implies that MMPs serve various functions in the context of ICH. Clinical studies have shown an independent association between baseline plasma MMP-9 levels and HG [65,66]. Higher elevated admission serum MMP-9 and MMP-3 levels have been found to be associated with PHE volumes, higher NIHSS scores and 90-day poor functional outcomes [67,68,69,70,71].

## 6. Potential Mechanisms

ICH-induced primary brain injury occurs several minutes after ictus and is due to mechanical disruption from hematoma, resulting in a sharp increase in intracranial pressure, even herniation. Secondary injury following ICH can activate the immune response. The inflammation pathway plays an essential role in exacerbating BBB disruption, mitochondrial dysfunction, neuronal apoptosis, worsened edema, and other pathophysiological consequences [10,42,55,72]. Following the initial brain injury, a cascade of inflammatory changes in the PHE tissue, composed of activated microglia and astrocytes and recruited leucocytes, propagates neuronal apoptosis, and axial degeneration. Crucially, secondary brain damage immediately develops and is mediated by cytotoxicity, excitotoxicity, the oxidative response, and the inflammatory effect of blood lysis [10,73]. Ferrous iron, a major hemoglobin degradation product, is remarkably neurotoxic through generating free radicals [74]. Emerging evidence shows that all these cascades of events result in poor outcomes for ICH. Theoretically, it would seem the most optimal to minimize the cascade of secondary injury and alleviate PHE as early as possible [38]. This concept is supported by animal experiments that have demonstrated that fingolimod might decrease brain edema, atrophy, and cell apoptosis and enhance recovery in ICH rats [75]. A recently published clinical study showed similar results [54,76].

## 7. Biomarker Panels

To more comprehensively address the complexity of the ICH cascade and enhance the accuracy of biomarkers as diagnostic tools, numerous studies have focused on biomarker panels rather than searching for a single biomarker. Montaner et al. [20] assessed plasma from 1015 patients (915 strokes and 90 mimics) for 10 serum biomarkers using an enzyme-linked immunosorbent assay in an emergency setting. They found that the independent predictors of stroke vs. mimics were caspase-3, D-dimer, soluble receptor for advanced glycation end products (sRAGE), chimerin, secretagogin, and MMP-9. Furthermore, when combining six biomarkers above or below their respective cut-off levels, this model demonstrated a predictive probability of stroke identification exceeding 99%. In a subsequent study conducted by this research team, they found that only S100B and sRAGE could effectively differentiate between acute ischemic stroke and ICH, achieving an area under the receiver operating characteristic curve of 0.76 for blood samples obtained within 3 h after the onset of symptoms [77]. A study published in 2016 with a small sample size found that RBP4 (>61 g/mL) and GFAP (<0.07 ng/mL) distinguished acute ischemic stroke from ICH with a specificity of 100% [16]. However, in a prospective multi-center study, the stroke-chip study, a panel of 21 biomarkers was assessed upon the immediate arrival of patients presenting within 6 h after symptom onset. None of these biomarkers demonstrated the ability to provide an ultra-early accurate differential diagnosis of stroke [17]. In recent decades, biomarkers have exhibited substantial promise for the diagnosis and prognosis of ICH. When integrated with pre-existing clinical and imaging data, they may provide additional information to help early treatment decision-making. From a clinical perspective, it is worth noting that serial biomarker measurements during the acute phases of ICH may offer increased diagnostic and predictive utility. Further validation remains necessary.

## 8. Surgical Intervention

Hematoma evacuation is widely regarded as a life-saving procedure for supratentorial ICH patients with brain herniation or midline shift [78]. Surgical decompression, whether with or without evacuation, may also potentially reduce mortality in supratentorial ICH patients presenting with moderate hematoma volumes. Surgical intervention after ICH offers the potential to mitigate secondary brain injury associated with the toxicity of hemoglobin degradation products. However, the result from a prospective randomized controlled trial, Surgical Trial in Intracerebral Hemorrhage (STICH I), indicated no significant advantage of surgical procedures compared with conservative treatment. Nevertheless, a subgroup analysis indicated the potential benefits of surgery in patients with lobar hematoma ≤1 cm from the cortical surface [79]. In light of these findings, the STICH II trial was conducted to evaluate the efficacy of early surgical intervention versus medical management in patients with lobar ICH volumes ranging from 10 to 100 mL without intraventricular hemorrhage [80]. There was no statistically significant difference in the rates of favorable outcome. Conventional craniotomy exhibits some drawbacks, including the risk of postoperative rebleeding and additional damage to surrounding brain tissue during surgery, all of which can contribute to increased mortality and morbidity. With the advancement of minimally invasive surgery (MIS), more recent reports have shown greater promise [81,82,83]. A recent meta-analysis of some randomized controlled trials (RCTs) demonstrated that MIS had a positive effect on outcomes compared with medical management and conventional craniotomy for ICH patients [84]. Minimally invasive surgery with thrombolysis in intracerebral hemorrhage evacuation (MISTIE III) was the first randomized controlled trial to investigate the performance of minimally invasive procedures in supratentorial ICH in association with functional outcomes [85]. The study found that patients who had a more significant hematoma reduction (residual hematoma ≤15 mL) had a higher likelihood of a good outcome at 1 year [86]. Furthermore, in the analysis of lobar ICH in MISTIE III and STICH II, end-of-treatment hematoma volume ≤28.8 mL in MISTIE III and ≤30.0 mL in STICH II showed an increased probability of modified Rankin Scale 0 to 3 at 180 days [87]. These findings imply that future advancements in surgical techniques aimed at residual hematoma volumes may yield even more promising results. Moreover, endoscopic evacuation is associated with a lower rate of rebleeding, a shorter hospital stay and the greatest improvement in mortality and functional independence [88,89]. In a retrospective study including 156 patients with spontaneous basal ganglia hemorrhage, it was observed that the mortality of the stereotactic aspiration group was significantly higher than that of the endoscopic aspiration group in the medium (≥40–<80 mL) and large (≥80 mL) hematoma subgroups [88]. Recent breakthroughs in therapeutic devices are significantly advancing the field of ICH treatment. Future studies should aim to develop predictive models or decision-support tools that incorporate both hematoma features and biomarker panels for determining the optimal management strategies for ICH.

## 9. Future Directions

Specifically, most studies reported on an association between blood biomarkers from single time-point samples after ICH onset and functional outcomes. This may lead to an improved utilization of time-limited treatments. However, as the concentrations of these biomarkers may peak several days after onset, analyzing dynamic changes at a single time point or merely up to 48 or 72 h after ictus may yield limited results. Using a single time point to evaluate a biomarker might not be sufficient to reflect the complex underlying mechanisms. Thus, the post-stroke dynamic changes in serum markers should be sampled at multiple time points. From a clinical perspective, it is crucial to note that sequential measurements of biomarkers are especially informative.

Given the importance of early management [90], considerable emphasis has been delegated to clinicians on making the strategy to expedite the early identification of high-risk patients. The identification of specific biomarkers might provide important information in individualizing the decision to initiate an anti-expansion treatment strategy. Although standardized assessment tools such as the ICH score and 24-point BRAIN score have been validated in clinical practice, these tests remain imperfect [91,92]. The inclusion of blood biomarkers in the prognostic model could be useful for patients with a predicted poor outcome and add key information in the early risk stratification.

## 10. Conclusions

**Figure 1:**
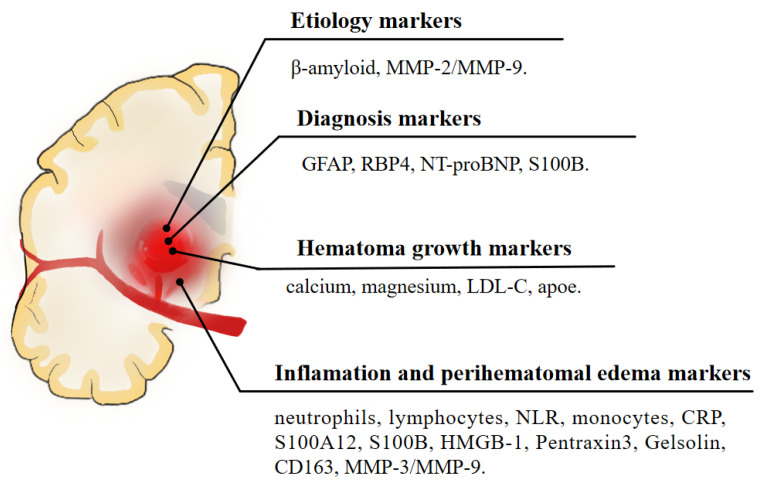
A schematic illustration of blood biomarkers of intracerebral hemorrhage.

The blood biomarkers for each category are summarized in Table 1 and Figure 1. Research on the identification of appealing blood markers that are related to ICH prognosis is developing at a rapid pace. Based on the existing studies, the research can be roughly divided into studies on brain edema, such as inflammatory biomarkers, oxidative stress biomarkers, neuron and astrocyte-specific markers, coagulation markers, genetic biomarkers, and metabolic biomarkers. Thus, it is possible that a biomarker or panel of biomarkers will be useful in providing prognostic information and determining individualized treatment decisions in the future. Further investigations with well-designed studies, large sample sizes, and comprehensive data will be needed to validate the findings.

Although research and clinical trials have questioned the availability of targeting inflammatory mediators in the therapy of ICH, the results remain controversial. This reality may be explained by the complex association between cell-mediated immune function and pro- and anti-inflammatory mediators. Thus, a comprehensive understanding of the functional role in the inflammatory reaction in ICH might help to develop and focus new therapeutic targets in the future.

## Figures and Tables

**Table 1:** Blood-based Biomarkers in Intracerebral Hemorrhage.

Blood Biomarker	Study Reference	Sample Size	Design	Time Point
**Diagnostic marker**				
GFAP and RBP-4	Mattila et al., 2021 [13]	272(60 ICH, 203 ischemic stroke, 9 stroke mimics)	Cohort study	<1.5 h after ictus
	Foerch et al., 2012 [14]	205 patients (39 ICH, 163 ischemic stroke, 3 stroke mimic)	Cohort study	<4.5 h after ictus
	Rozanski et al., 2017 [15]	74 patients (25 ICH, 49 ischemic stroke)	Cohort study	-
	Llombart et al., 2016 [16]	144 patients	Cohort study	<6 h after ictus
NT-proBNP	Bustamante et al., 2017 [17]	1308 patients	Multi-center study	<6 h after ictus
	Bustamante et al., 2021 [18]	189 patients (154 with ischemic stroke and 35 with ICH)	Cohort study	<4.5 h after ictus
S100B	Montaner et al., 2012 [20]	337 patients	Cohort study	<6 h after ictus
**Etiology marker**				
β-amyloid	Guillamon et al., 2012 [21]	29 ICH patients; 21 healthy controls	Case control	<6 h after ictus
MMP-2 AND MMP-9	Guillamon et al., 2012 [23]	48 CAA-associated hemorrhagic patients; 21 controls	Case control	<3 days after ictus
**Hematoma growth marker**				
Calcium	Tu et al. 2019 [24]	1262 patients	Cohort study	<12 h after ictus
	Zhang et al., 2018 [25]	111 patients	Cohort study	<6 h after ictus
	Elkhatib et al., 2019 [26]	137 patients	Cohort study	<12 h after ictus
	Sallinen et al., 2019 [27]	433 patients	Cohort study	<72 h after ictus
	Morotti et al., 2016 [28]	2103 patients	Cohort study	<72 h after ictus
Magnesium	Liotta et al., 2017 [29]	290 patients	Cohort study	-
	Goyal et al., 2018 [30]	299 patients	Cohort study	<24 h after ictus
LDL-C	Rodriguez et al., 2011 [32]	108 patients	Cohort study	<6 h after ictus
	Chang et al., 2018 [31]	672 patients	Cohort study	<24 h after ictus
	Elkhatib et al., 2019 [26]	137 patients	Cohort study	<12 h after ictus
	Wang et al., 2022 [34]	73098 patients	Cohort study	<7 days after ictus
Apolipoprotein E	Brouwers et al., 2012 [35]	510 patients	Cohort study	<48 h after ictus
**Inflammatory or PHE marker**				
Neutrophils	Kayhanian et al., 2017 [41]	113 patients	Cohort study	-
	Morotti et al., 2016 [11]	1302 patients	Cohort study	<48 h after ictus
NLR	Lattanzi et al., 2016 [12]	177 patients	Cohort study	<24 h after ictus
	Giede-Jeppe et al., 2017 [44]	855 patients	Cohort study	-
	Qin et al., 2019 [45]	213 patients	Cohort study	<24 h after ictus
	Gusdon et al. 2017 [46]	153 patients	Cohort study	<24 h after ictus
	Mackey et al., 2021 [47]	593 patients	Cohort study	<24 h after ictus
	Yu et al., 2016 [48]	2630 patients	Cohort study	<6 h after ictus
Monocyte	Morotti et al., 2016 [11]	1302 patients	Cohort study	<48 h after ictus
	Mackey et al., 2021 [47]	593 patients	Cohort study	<24 h after ictus
	Adeoye et al., 2014 [49]	186 patients	Cohort study	<12 h after ictus
	Walsh et al., 2015 [50]	240 patients	Cohort study	<24 h after ictus
CRP	Alexandrova et al., 2011 [52]	46 patients	Cohort study	-
	Löppönen et al., 2014 [53]	807 patients	Cohort study	<24 h after ictus
	Napoli et al., 2012 [8]	223 patients	Cohort study	<24 h after ictus
	Napoli et al., 2011 [54]	210 patients	Cohort study	<24 h after ictus
S100A12	Qiu et al., 2021 [9]	101 patients	Cohort study	<24 h after ictus
	Qian et al., 2018 [58]	182 ICH patients; 182 healthy controls	Case control	<24 h after ictus
S100B	Hu et al., 2010 [59]	86 ICH patients; 30 healthy controls	Case control	<6 h after ictus
	Delgado et al., 2006 [19]	78 ICH patients	Cohort study	<24 h after ictus
HMGB-1	Zhou et al., 2010 [60]	65 ICH patients; 41 healthy controls	Case control	<12 h after ictus
Pentraxin 3	Hao et al. 2021 [61]	307 ICH patients; 132 healthy controls	Case control	<12 h after ictus
Gelsolin	Zhao et al. 2013 [62]	132 ICH patients; 68 healthy controls	Case control	<6 h after ictus
CD163	Roy-O’Reilly 2017 [64]	51 ICH patients; 10 healthy controls; 24 TIA patients	Case control	<48 h after ictus
MMP-9	Yang et al. 2016 [65]	186 patients	Cohort study	<12 h after ictus
	Silva et al. 2005 [66]	183 patients	Cohort study	<12 h after ictus
	Castellazzi et al.,2010 [67]	28 patients	Cohort study	<24 h after ictus
	Abilleira et al., 2003 [71]	57 patients	Cohort study	<24 h after ictus
MMP-3 AND MMP-9	Howe et al., 2019 [68]	55 patients	Cohort study	-
	Alvarez-Sabín et al., 2004 [69]	21 patients	Cohort study	<12 h after ictus
	Li et al., 2013 [70]	59 patients	Cohort study	<24 h after ictus

GFAP: glial fibrillary acidic protein; RBP-4: retinol-binding protein; NT-proBNP: N-Terminal Pro B-Type Natriuretic Peptide; CAA: cerebral amyloid angiopathy; LDL-C: Low-density lipoprotein cholesterol; PHE: perihematomal edema; NLR: neutrophil–lymphocyte ratio; CRP: C-reactive protein; HMGB-1: High Mobility Group Box-1; MMP: matrix metalloproteinases.

## Data Availability

Not applicable.
